# Chronic and Acute Drug-Induced Hypersensitivity Syndrome in a Rural Patient With Cytomegalovirus Infection: A Case Report

**DOI:** 10.7759/cureus.61376

**Published:** 2024-05-30

**Authors:** Saaya Nakazato, Shota Ogawa, Kohei Oka, Chiaki Sano, Ryuichi Ohta

**Affiliations:** 1 Family Medicine, Shimane University Faculty of Medicine, Izumo, JPN; 2 Community Care, Unnan City Hospital, Unnan, JPN; 3 Community Medicine Management, Shimane University Faculty of Medicine, Izumo, JPN

**Keywords:** rural, general medicine, family medicine, lymphocyte stimulation test, prednisolone, ampicillin, cytomegalovirus infections, lymphadenopathy, drug-induced hypersensitivity syndrome

## Abstract

A 50-year-old man presented with fever and a generalized rash, with chronic fatigue and lymphadenopathy for a year and a half. Initial tests ruled out lymphoproliferative disorders, showing reactive hyperplasia and cytomegalovirus. Symptoms worsened after ampicillin treatment, leading to suspected drug-induced hypersensitivity syndrome (DIHS). Upon admission, amoxicillin was discontinued, and prednisolone and antiviral treatment were initiated. The patient's condition improved with this therapy. A drug-induced lymphocyte stimulation test confirmed hypersensitivity to both ampicillin and allopurinol. This case illustrates the diagnostic challenge of chronic and acute DIHS because of the rare presentation. It underscores the need for high suspicion of DIHS in patients with chronic lymphadenopathy and fatigue, particularly with recent drug exposure. Effective management involves recognizing symptoms, withdrawing the offending drug, and using corticosteroids. Viral infections like cytomegalovirus can complicate DIHS diagnosis and treatment, necessitating a comprehensive approach. This case highlights the importance of considering DIHS in differential diagnoses and the complexities of managing it alongside co-infections in rural healthcare settings.

## Introduction

Drug-induced hypercreativity syndrome (DIHS) is a syndrome triggered by a type 4 allergy to specific drugs [1]. DIHS can typically show acute symptoms 7 to 10 days after starting suspected drugs, such as fever, rash, liver enzyme elevation, and high inflammatory conditions [2]. Diagnosis of DIHS can be determined by clinical symptoms and drug-induced lymphocyte stimulation test (DLST) [3]. The treatments are the withdrawal of the suspected drugs, and in critical cases, prednisolone can be used in the short term [4]. Clinicians should be keen on various symptoms several weeks after starting a new medicine.

DIHS can cause chronic symptoms such as fatigue and systemic lymphadenopathy. As the symptoms are mild, patients may not notice such symptoms triggered by a specific medicine [5]. Investigating specific lymph nodes can rule out malignant diseases, but it is challenging to differentiate DIHS and other inflammatory diseases [6]. Patients with chronic DIHS can have hypersensitivity to another drug, which can trigger acute DIHS symptoms. This time, we experienced a 50-year-old male suffering from chronic systemic lymphadenopathy, eventually diagnosed with chronic DIHS by allopurinol and acute DIHS by ampicillin, coexistence with cytomegalovirus infection. Through this case, we discuss the difficulty of diagnosing chronic DIHS and specific pathophysiology and strategies for acute and chronic DIHS in rural contexts.

## Case presentation

**Figure 1:**
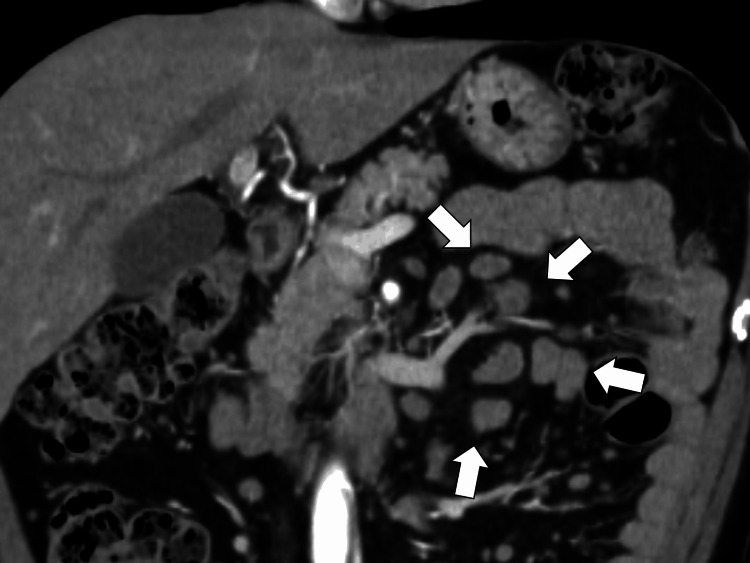
Abdominal computed tomography showing localized abdominal lymphadenopathy (white arrows)

A 50-year-old man presented to a rural community hospital with the chief complaints of fever and generalized skin rash. He had had general fatigue for one and a half years ago, and abdominal computed tomography (CT) showed localized abdominal lymphadenopathy (Figure 1).

He had been investigated for a lymphoproliferative disorder. A mesenteric lymph node biopsy showed no amyloid deposits or atypical lymphocytes and was diagnosed as reactive hyperplasia. The results from duodenal and colon biopsies also showed no signs of amyloidosis. The biopsy of the duodenum showed the presence of cytomegalovirus. However, no progressive inflammation and negative results of cytomegalovirus antigenemia did not demand cytomegalovirus treatment, considering the side effects of the treatment. His fatigue continued, and he was observed in outpatient departure.

Twelve days before admission, he had a sore throat and mild fever, was diagnosed with viral pharyngitis, and was prescribed acetaminophen at a primary care clinic. As his symptoms got worse, he visited an urban general hospital 10 days before admission. He was admitted to the general hospital with the diagnosis of bacterial pharyngitis and treated with intravenous ampicillin. Four days after the treatment, he was discharged with an amoxicillin prescription. One day before the admission to our rural hospital, he had a fever of 39.0 °C and a generalized skin rash spread from the trunk to the extremities. He came to our hospital for an investigation. His past medical history included dyslipidemia, gastroesophageal reflux disease, and hyperuricemia. His medication history included atorvastatin of 10 mg, esomeprazole of 20 mg, and allopurinol of 100 mg daily for more than 10 years.

**Figure 2:**
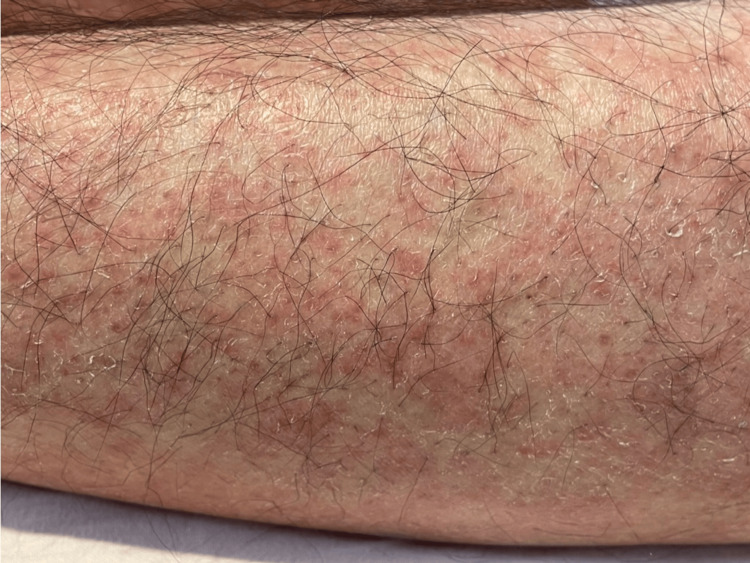
Erythema and petechiae on the left lower leg

On the day of the admission, consciousness was clear regarding time, place, and person. The vital signs were as follows: body temperature, 39.0 °C; pulse, 135 bpm; blood pressure, 118/81 mmHg; respiratory rate, 22 times/minute; and SpO2, 99%. Physical examination showed generalized erythema and petechiae observed all over the body (Figure 2).

Pain in the posterior neck and head, bilateral costal pain, and upper abdominal tenderness were noted. Posterior cervical and submandibular lymph nodes were palpable. The initial laboratory test showed an elevated C-reactive protein (Table 1).

**Table 1:** Initial laboratory data of the patient AU, arbitrary unit; EBV, Epstein-Barr virus; CMV, cytomegalovirus; CRP, C-reactive protein; Ig, immunoglobulin; VCA, virus capsid antigen

Parameter	Level	Reference
White blood cells	9.4× 10^3^	3.5–9.1 × 10^3^/μL
Neutrophils	86.0	44.0–72.0%
Lymphocytes	11.3	18.0–59.0%
Eosinophil	2.1	< 5%
Hemoglobin	13.1	11.3–15.2 g/dL
Hematocrit	39.3	33.4–44.9%
Mean corpuscular volume	88.9	79.0–100.0 fl
Platelets	22.9 × 10^4^	13.0–36.9 × 10^4^/μL
Total protein	7.8	6.5–8.3 g/dL
Albumin	3.7	3.8–5.3 g/dL
Total bilirubin	0.5	0.–1.2 mg/dL
Aspartate aminotransferase	15	8–38 IU/L
Alanine aminotransferase	14	4–43 IU/L
Lactate dehydrogenase	144	121–245 U/L
Blood urea nitrogen	15.7	8–20 mg/dL
Creatinine	1.05	0.40–1.10 mg/dL
Serum Na	134	135–150 mEq/L
Serum K	4.5	3.5–5.3 mEq/L
Serum Cl	97	98–110 mEq/L
Ferritin	69.6	14.4–303.7 ng/mL
CRP	2.2	<0.30 mg/dL
IgG	1366	870–1700 mg/dL
IgM	160	35–220 mg/dL
IgA	504	110–410 mg/dL
CMV-IgM	<0.1	<0.1
CMV-IgG	220 AU/mL	<6.0 AU/mL
EBV anti-VCA-IgM	<10 times	<10 times
EBV anti-VCA-IgG	80 times	<10 times
Urine test	-	-
Leukocyte	Negative	Negative
Protein	Negative	Negative
Blood	Negative	Negative

**Figure 3:**
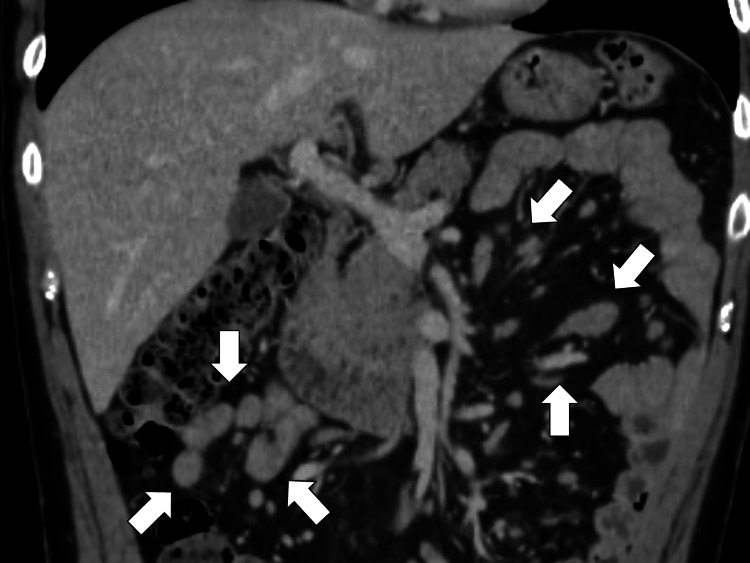
Abdominal computed tomography showing multiple enlarged lymph nodes in the mesentery (white arrows)

Abdominal CT showed multiple enlarged lymph nodes in the mesentery (Figure 3).

Upon admission, amoxicillin, suspected to be the cause of the drug eruption and drug-induced hypersensitivity syndrome (DIHS), was discontinued.

On the second day of hospitalization, his fever was persistent at over 39 °C, and his appetite was progressive. Considering the progression of DIHS, prednisolone of 30 mg per day was started. Considering the patient's hospitalization history within the past month, the possibility of septic erythema and purpura could not be excluded. Piperacillin and tazobactam (PIPC/TAZ) of 13.5 g intravenously were thus started suspecting sepsis. On the fourth day of hospitalization, his general malaise was progressive, and his vital signs showed tachycardia of 110 times per minute and tachypnea of 24 times per minute; cytomegalovirus reactivation and acute exacerbation were suspected. The treatment with oral valganciclovir of 900 mg per day was initiated. On the same day, the dose of prednisolone was increased to 60 mg for increasing intensity of the treatment of DIHS.

**Figure 4:**
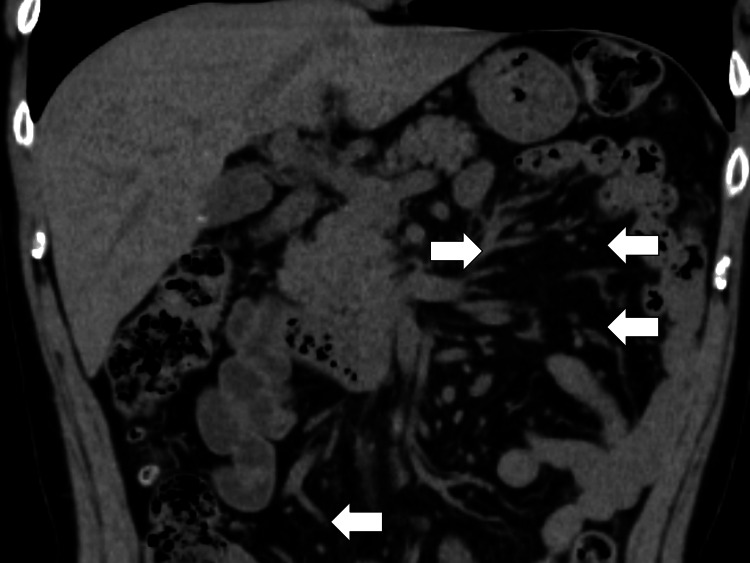
Follow-up abdominal computed tomography showing the decrease in swollen lymph nodes in the mesentery (white arrows)

Allopurinol, which he was taking orally, could cause a similar rash, so it was discontinued. On the fifth day, his body temperature dropped to the 36 °C range, and by the sixth day, the rash had lessened. Blood cultures were negative on the seventh day, so PIPC/TAZ was discontinued on the seventh day. The rash disappeared by the seventh day. On the eighth day of admission, valganciclovir was stopped. Prednisolone was tapered gradually by 10 mg weekly with no relapse observed. On the tenth day of admission, he was discharged to his home. On outpatient department follow-up, a drug-induced lymphocyte stimulation test (DLST) was performed for ampicillin and allopurinol, and the results were positive for both drugs. Two weeks after the discharge, his abdominal CT showed a decrease in swollen lymph nodes in the mesentery (Figure 4).

## Discussion

This case illustrates a middle-aged man with chronic lymphadenopathy and multiple symptoms suggestive of DIHS, ultimately diagnosed with both acute and chronic DIHS modified by cytomegalovirus infection. This case emphasizes the complexity and diagnostic challenges of DIHS, particularly in rural settings where access to specialized diagnostic tools and healthcare providers may be limited.

As evidenced in this case, DIHS can manifest with chronic symptoms such as persistent fatigue and systemic lymphadenopathy. Previous research has indicated that chronic DIHS can be overlooked due to its subtle clinical presentation, often leading to misdiagnosis or delayed diagnosis [7,8]. This patient’s prolonged fatigue and generalized lymphadenopathy, initially investigated for lymphoproliferative disorders, underscore the need for a high index of suspicion for DIHS in similar clinical scenarios [9]. General physicians should recognize that DIHS can have various presentations and should be included in the differential diagnosis of vague symptoms in primary care settings [10].

The coexistence of cytomegalovirus infection complicated the diagnosis of DIHS in this patient. Cytomegalovirus reactivation can mimic or exacerbate symptoms of DIHS, as seen in this case where the patient presented with fever, rash, and worsening fatigue [11]. Previous studies have shown that viral infections can act as cofactors, worsening the clinical course of DIHS and complicating its management [12]. In this context, initiating valganciclovir alongside corticosteroids was crucial in controlling the DIHS and the viral infection, facilitating a smoother remission of the patient’s inflammatory symptoms [4,13]. Systemic lymphadenopathy in acute and chronic phases demands the investigation of viral infections such as cytomegalovirus infections [14]. In critical cases, antivirus treatments should be started promptly to prevent exacerbation.

Effective treatment of DIHS, mainly when systemic symptoms are severe, often involves the use of corticosteroids such as prednisolone [15]. In this case, the administration of prednisolone significantly improved the patient's symptoms. The gradual tapering of prednisolone, once the acute symptoms are controlled, is in line with recommendations to minimize potential side effects associated with long-term steroid use [1]. Additionally, discontinuation of suspected causative drugs, including amoxicillin and allopurinol, was necessary to prevent further hypersensitivity reactions [16]. The coexistence of virus infections may avoid immunosuppressant usage, but the acute exacerbating phase of DIHS should underscore prednisolone usage to treat DIHS effectively.

DLST, which confirmed hypersensitivity to ampicillin and allopurinol, further supported the diagnostic process in this case. Previous studies have validated DLST as a valuable tool for identifying specific drug hypersensitivities and guiding appropriate management and prevention strategies [17]. However, in this case, the result of DLST was delayed because this test was not performed outside of our prefecture. The limited availability and delayed results of DLST in rural settings pose a challenge, highlighting the need for increased accessibility to specialized diagnostic services in these areas [18]. In addition, the cutoff of DLST can differ in countries, which may confuse medical professionals in assessing type four allergies to medications [19]. Even if DLST is negative, there is a possibility that one medicine can be a cause of DIHS. DLST can be used just for one reference of the diagnosis of DLST. Rural physicians should suspect DIHS clinically when the other differential diagnoses are ruled out and patients use suspicious medications [20].

## Conclusions

This case highlights the importance of considering DIHS in patients presenting with unexplained chronic lymphadenopathy and fatigue, particularly when there is a history of recent drug exposure. It also underscores the need for comprehensive diagnostic evaluations, including the use of DLST and consideration of viral cofactors such as cytomegalovirus. The effective management of DIHS involves prompt recognition, withdrawal of the offending drug, and appropriate use of corticosteroids, with attention to potential co-infections that may complicate the clinical course.
